# Management of Locally Advanced Esthesioneuroblastoma in a Pregnant Woman

**DOI:** 10.1155/2019/3789317

**Published:** 2019-08-19

**Authors:** Inês Maria Guerreiro, Cláudia Vieira, André Soares, António Braga, Manuel Jácome, José Dinis

**Affiliations:** ^1^Medical Oncology Department, Instituto Português de Oncologia do Porto Francisco Gentil (IPO-PORTO), Porto, Portugal; ^2^Radiation Oncology Department, Instituto Português de Oncologia do Porto Francisco Gentil (IPO-PORTO), Porto, Portugal; ^3^Department of Obstetrics and Gynecology, Centro Hospitalar do Porto, Porto, Portugal; ^4^Pathology Department, Instituto Português de Oncologia do Porto Francisco Gentil (IPO-PORTO), Porto, Portugal

## Abstract

Esthesioneuroblastoma (ENB) is a rare malignant tumor that commonly develops in the upper nasal cavity. Standard treatment is not established, especially in locally advanced disease which portends the worse prognosis. Hereby, we report a case of a 27-year-old, 23-week pregnant woman, with a 2-month history of progressively growing right cervical lymphadenopathy, nasal obstruction, anosmia, frequent episodes of epistaxis, and right frontal headache. Imagiological evaluation revealed a lesion with 7×5,2×3,2 cm in the nasal fossae with extension to the ethmoidal complex and right olfactive fend and invasion of the endocranial compartment associated with lymphadenopathy. The biopsy revealed a high-grade EBN. Neoadjuvant chemotherapy with cisplatin and etoposide was administrated during pregnancy and continued after delivery up to 6 cycles of treatment with partial response. Radiotherapy followed, with complete response. This case report is intended to highlight that a high grade of suspicion should be kept in the presence of nonspecific symptoms of nasal obstruction, anosmia, facial pain, and/or headache and focus that chemotherapy is an important component of a combined-treatment modality for locally advanced ENB that can be used during pregnancy in a lifesaving situation.

## 1. Background

**Figure 1:**
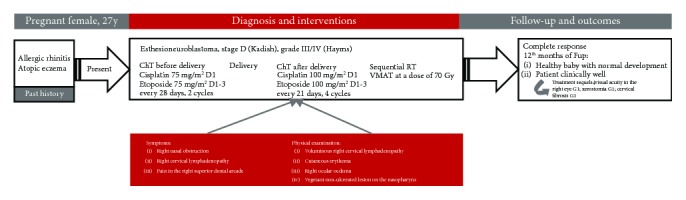
Timeline.

Esthesioneuroblastoma (ENB), also referred to as olfactory neuroblastoma (ONB), is a malignant neuroectodermal tumor thought to originate from the olfactory membrane of the sinonasal tract [1]. ENB is a rare tumor and most commonly develops in the upper nasal cavity in the region of the cribriform plate [1]. Consequently, the most common symptom is nasal obstruction that can be associated with epistaxis and pain [1–3]. Manifestations of locally advanced disease include anosmia, proptosis, facial pain, or frontal headache due to invasion of adjacent structures [2]. There is a slight male predominance and a bimodal age presentation in the second and sixth decade of life [1, 2]. The best treatment options in advanced stages are not well defined due to the rarity of the disease. The most frequent staging system is the Kadish staging modified by Morita and colleagues [4]. A cervical lymph node or distant metastases represent the most advanced stage of the disease—stage D. The grading system developed by Hyams et al. [5] classifies tumors in 4 groups based on mitotic activity, nuclear pleomorphism, rosette formation, necrosis, and the characteristics of the fibrillary matrix. Both extent of disease and grading appear to have a prognostic significance [6, 7]. We report the case of a pregnant woman with a locally advanced esthesioneuroblastoma (Figure 1).

## 2. Case Presentation

**Figure 2:**
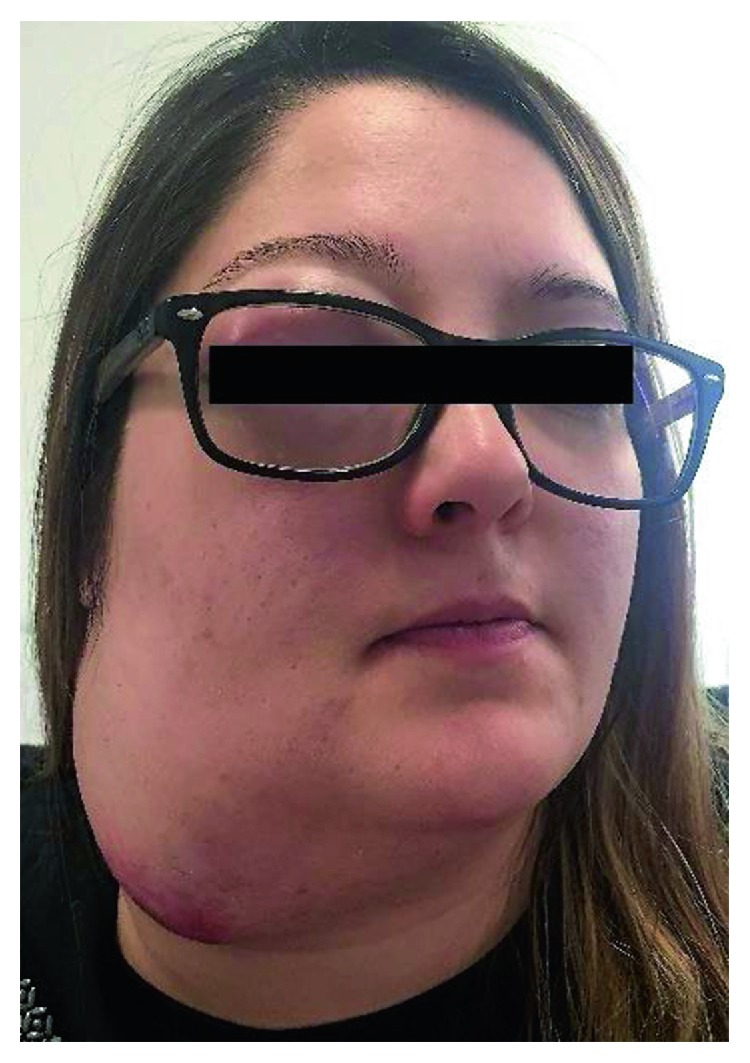


**Figure 3:**
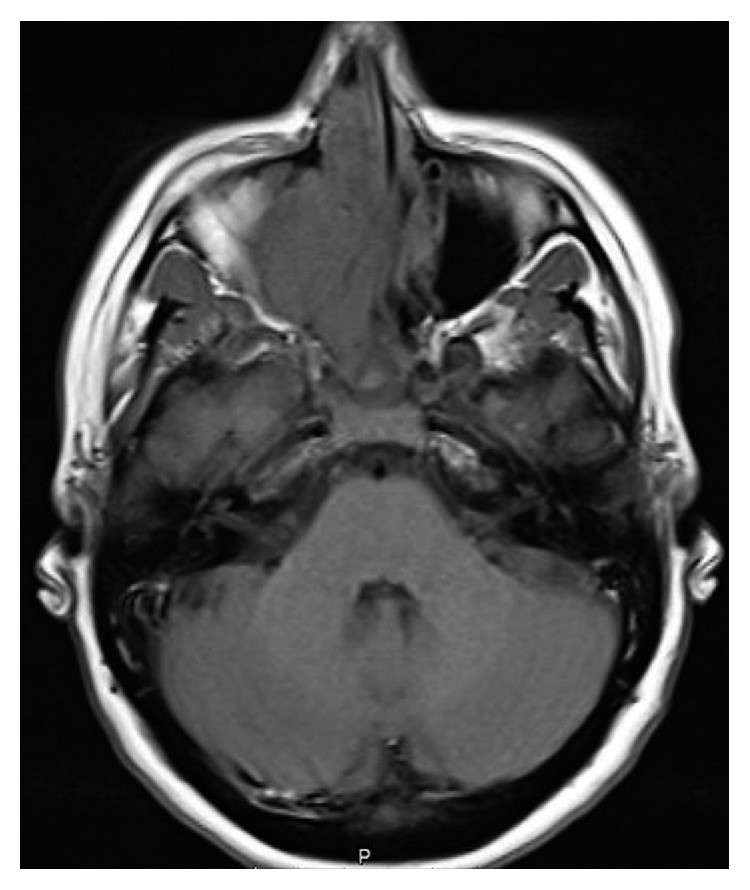


**Figure 4:**
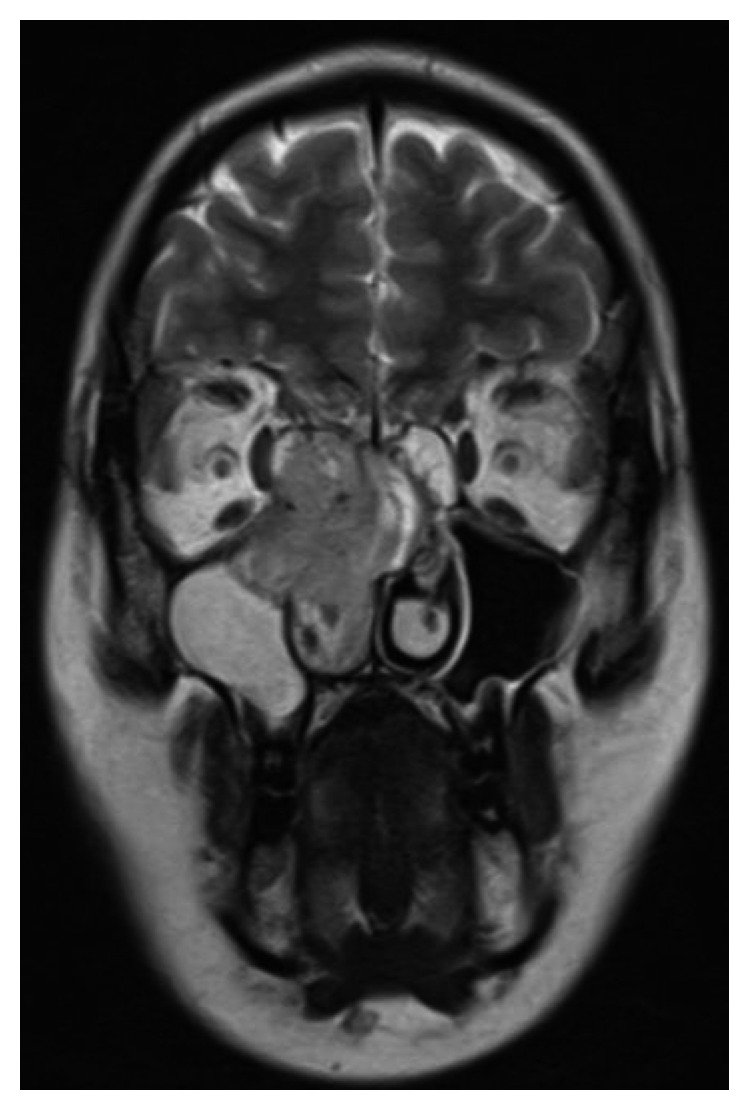


A 27-year-old 8-week pregnant woman, with a history of allergic rhinitis and atopic eczema, presented to a general hospital with right nasal obstruction, right cervical lymphadenopathy, and pain in the right superior dental arcade. A nonsteroidal anti-inflammatory drug was started with resolution of the dental pain after one week of treatment. Two months later, while maintaining a progressively growing right cervical lymphadenopathy and right nasal obstruction, the patient developed anosmia, frequent episodes of epistaxis, and right frontal headache. A fine needle biopsy of the lymphadenopathy was performed with an inconclusive result, revealing only the presence of inflammatory cells. A core biopsy was then performed which revealed lymph node metastasis from a poorly differentiated malignant neoplasm. At the 23rd week of pregnancy, the patient was referred to our hospital. On physical examination, the patient had a voluminous right cervical lymphadenopathy with 15 cm from levels Ib to V associated with cutaneous erythema as well as right ocular oedema (Figure 2). A vegetant nonulcerated lesion was detected on the nasopharynx occupying the right nasal vestibulum. A biopsy of the lesion was performed. Pathology's result revealed respiratory epithelium with focal involvement by small round blue cells, neuron-specific enolase (NSE) positive, synaptophysin positive, PS 100 positive, and AE1/AE3 and CD99 negative. The cranial and cervical magnetic resonance images (MRI) revealed a lesion with 7×5,2×3,2 cm in the nasal fossae, ethmoidal complex, and right olfactive fend with invasion of the endocranial compartment and the orbit and deviation of the internal rectum muscle as well as extension to the nasopharynx lumen and invasion of the sphenoidal sinus associated with lymphadenopathy in the retropharyngeal area and right II, III, IV, and V levels (Figures 3 and 4). The patient was diagnosed with a right esthesioneuroblastoma stage D in the modified Kadish grading system [4] and grade III/IV in the Hyams grading system [5].

The case was evaluated by a multidisciplinary team of head and neck surgeons, medical oncologists, and radiation oncologists. The multidisciplinary tumor board determined that there was no indication to perform surgery due to local extent of the disease. The patient was proposed to do systemic treatment with chemotherapy followed by reevaluation by the multidisciplinary tumor board. Treatment with cisplatin 75 mg/m^2^ on day 1 and etoposide 75 mg/m^2^ on days 1 to 3, cycles every 28 days, was started after an appropriate discussion with the patient's obstetrician. The following premedication before each treatment cycle was prescribed: hydrocortisone 100 mg, metoclopramide 10 mg, and ondansetron 8 mg. Additional treatment with daily folic acid, oral iron, iodine supplementation, and prophylactic enoxaparin was made as recommended by the obstetrician.

**Figure 5:**
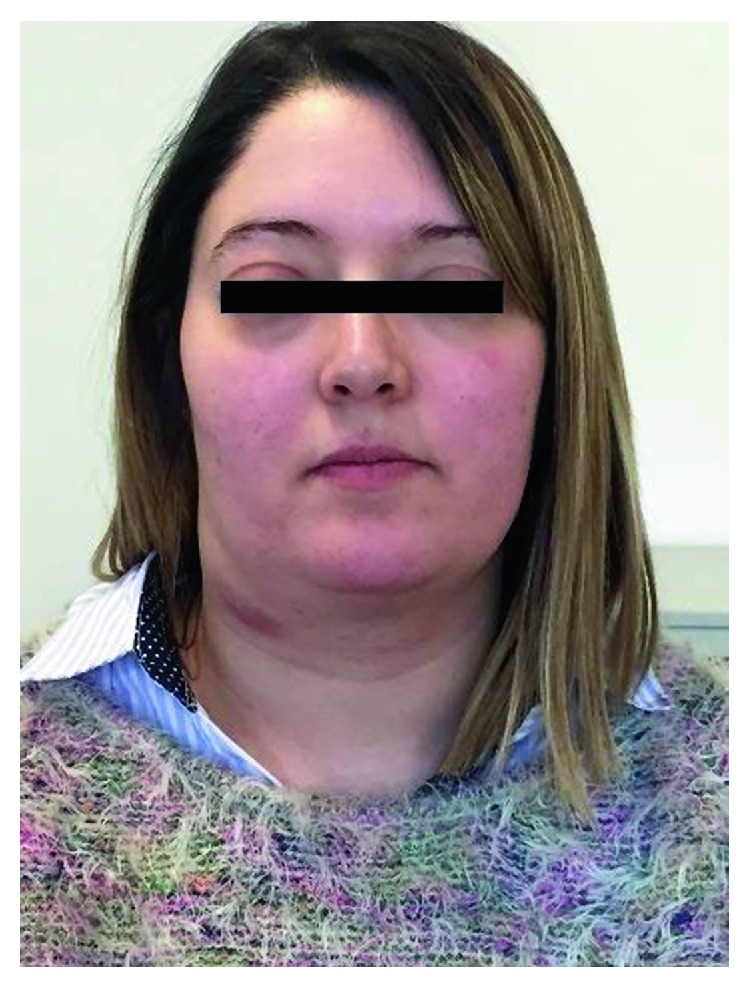


After the first cycle of treatment, a clinical reduction of the lesion was noted (Figure 5). Concerning the baby development, routine amniotic fluid assessment made by foetal echography after the 2nd cycle of chemotherapy showed an increase in systolic velocity in the Doppler midfoetal cerebral artery (systolic peak > 1.5 MoMs for gestational age). This finding was in favour with an established foetal anemia and interpreted as a side effect of chemotherapy. Once the foetus was stable (normal foetal biophysical profile), a foetal lung maturation cycle with betamethasone was performed according to protocol, and a decision to terminate the pregnancy in an elective manner was made, thus avoiding the worsening of the condition with a new cycle of chemotherapy. In addition, after 30 weeks of gestation, clinical suspicion of foetal anemia is an indication for termination of pregnancy, avoiding invasive foetal studies. Thus, 21 days after the second cycle of treatment and at 31 weeks of pregnancy, the patient delivered by caesarean a healthy baby uneventfully. While hospitalized, the new-born presented a normal development and no health problems were detected.

Fourteen days after the delivery, chemotherapy was resumed at a full dose with cisplatin 100 mg/m^2^ on day 1 and etoposide 100 mg/m^2^ on days 1 to 3, cycles every 21 days. After 4 cycles of treatment, a positron emission tomography-computed tomography (PET-CT) and MRI were performed, revealing a partial response. The patient completed 6 cycles of treatment with good tolerance. The main toxicities reported during treatment were grade 1 anemia, grade 1 nausea, and emesis treated with oral iron, folic acid, and metoclopramide as needed.

The case was again discussed in a multidisciplinary tumor board, and treatment with radiotherapy (RT) was proposed. The patient performed 33 fractions of treatment with volumetric modulated arc therapy (VMAT) at a dose of 70 Gy to the neoplastic lesion, right retropharyngeal area, and right cervical Ib to V levels and 50 Gy to the left retropharyngeal area, left cervical Ib to V levels, and left perinasal area. The treatment had a duration of 45 days with good tolerance. The main toxicities were grade 2 dysphagia, grade 2 odynophagia, grade 2 xerostomia, grade 2 oral mucositis, and grade 2 cervical dermatitis.

A period of clinical vigilance was started, and 12 weeks after the last treatment of radiotherapy, a PET-CT was performed revealing no radiopharmaceutical uptake. At the 12th month of follow-up, the baby is healthy and presents a normal development. The patient is clinically well presenting as treatment sequels a grade 1 diminution of visual acuity in the right eye, xerostomia grade 1, and cervical fibrosis grade 1.

## 3. Conclusions

This clinical case demonstrates the challenge of diagnosis and management of a rare and aggressive type of esthesioneuroblastoma in a pregnant woman. ENB is an uncommon tumor that frequently presents with nonspecific symptoms such as nasal obstruction and facial pain. The diagnosis is a challenge for the clinicians due to the rarity and unspecificity of this entity. Moreover, while the histologic diagnosis of a well-differentiated tumor is quite evident, a less differentiated form can be more laborious requiring additional markers for the differential diagnosis among the group of small round cell malignant neoplasms that can affect the sinonasal tract. Olfactory neuroblastomas frequently express NSE and synaptophysin and are negative for the expression of leucocyte common antigen and CD99 [1]. The entities to be considered in differential diagnosis are neuroendocrine carcinomas, melanoma, rhabdomyosarcoma, sinonasal undifferentiated carcinoma, squamous cell carcinoma (including NUT carcinoma), lymphoma, Ewing sarcoma, and metastatic tumors [1, 8]. Both the nonspecific presentation and the rare histological diagnosis contribute to a delay for the final diagnosis which justifies that the majority of the ENB are found in an advanced stage [9] as was the case in the clinical case described.

The standard treatment for ENB has not been established. The lack of prospective data due to its rarity and the absence of molecular therapeutic targets are contributing factors [10, 11]. Surgery followed by RT has been the most frequent treatment strategy and is associated with improved overall survival compared with surgery or RT alone [12, 13]. Patients with cervical lymph node metastasis at presentation have a worse prognosis despite combined-treatment modality [13], and the optimal management of these patients is uncertain. This clinical case describes a 23-week pregnant woman with a stage D ENB with cervical lymph node metastasis from levels Ib to V. The aggressive phenotype of the disease characterized by a painful and rapidly growing locally advanced tumor with lymph node metastasis and local invasion of the orbit and endocranial compartment motivated the decision to do treatment with neoadjuvant chemotherapy followed by radiotherapy. In fact, there is some published data that suggests that EBN is chemosensitive, and the addition of induction chemotherapy to a combined-treatment modality in patients with advanced ENB may improve treatment outcomes [10, 13]. The objective response to chemotherapy varied between 68% and 82% [10, 13–15], with a 5-year overall survival rate achieving 78% [13]. Various chemotherapy agents have been used, being cisplatin-based regimens (notably cisplatin and etoposide) frequently chosen with encouraging responses [16]. In what concerns the treatment strategy, the 23-week pregnancy of the patient posed an increasing challenge since the diagnosis of cancer during pregnancy is uncommon [17], and data about chemotherapy in esthesioneuroblastoma during pregnancy is missing.

Exposure to some chemotherapeutic agents during the second and third trimesters has not been associated with increased risk of foetal malformations or major problems in the short and long terms [17–19], contrarily to administration of chemotherapy during the first trimester of gestation which is associated with an increased risk of foetal congenital defects [17]. Cisplatin is usually safe for the foetus in the second and third trimesters [20] for doses up to 75 mg/m^2^ every 3 weeks [17]. There is evidence [21] that the rate of malformations after exposure to cisplatin in the first trimester is about 20% but is reduced to 1% in the second and third trimesters of pregnancy. Regarding etoposide, the data is more limited reporting 3% of major malformations during the second and third trimesters of pregnancy and a foetal growth restriction in 24% of cases [21].

Considering that it was a lifesaving situation for both the pregnant patient and her baby, the decision was to start treatment with chemotherapy with a regimen of cisplatin and etoposide that was administered during the 24th to 31st week of pregnancy. Furthermore, due to the high emetogenic potential of cisplatin-based regimens, it was essential to decide the pretreatment associated with each cycle of chemotherapy for prevention of chemotherapy-induced nausea and vomiting in a pregnant patient. Both the dopamine D2 antagonist metoclopramide and the serotonin 5-hydroxytryptamine type (5-HT3) receptor antagonist ondansetron can be used during pregnancy as there is no report of an increased risk of pregnancy-related events [22]. In relation to corticosteroids, there is an association between oral clefts and use of systemic steroids during the first trimester of pregnancy, so its use should be cautious before 10 weeks of gestation [22]. Outside the first trimester of pregnancy, systemic corticosteroids can be used at the lowest effective dose and for the shortest duration of time.

There were no complications during the neonatal period and the 12th month period of follow-up for both the baby and the mother, although a longer period of follow-up is necessary to ascertain this good outcome. Due to the aggressive nature of the esthesioneuroblastoma, clinical evaluation is made on a monthly basis.

This case highlights that chemotherapy is an important component of the multimodal treatment strategy of locally advanced esthesioneuroblastoma and shows that in a lifesaving situation, chemotherapy with cisplatin and etoposide can be administered in the 2nd and 3rd trimesters of pregnancy. Moreover, a prompt diagnosis is critical to achieve complete remission of the disease especially in the case of a biologically aggressive neoplasm. Prognosis of patients diagnosed with cancer during pregnancy is usually comparable with nonpregnant patients [17]; however, since recurrence of this type of tumor may develop 10 or more years following initial treatment [23, 24], a prolonged surveillance is mandatory.

This clinical case should be looked as a contribution to future similar cases as it is a unique illustration of a rare and aggressive esthesioneuroblastoma in a pregnant woman.

## Abbreviations

ENB: Esthesioneuroblastoma
MRI: Magnetic resonance image
NSE: Neuron-specific enolase
OBN: Olfactory neuroblastoma
PET-CT: Positron emission tomography-computed tomography
RT: Radiotherapy
VMAT: Volumetric modulated arc therapy.
